# Restriction of Viral Glycoprotein Maturation by Cellular Protease Inhibitors

**DOI:** 10.3390/v16030332

**Published:** 2024-02-22

**Authors:** Rishikesh Lotke, Moritz Petersen, Daniel Sauter

**Affiliations:** Institute for Medical Virology and Epidemiology of Viral Diseases, University Hospital Tübingen, 72076 Tübingen, Germany

**Keywords:** viral glycoprotein, protease, protease inhibitor, furin, TMPRSS2, serpins, antiviral host factors

## Abstract

The human genome is estimated to encode more than 500 proteases performing a wide range of important physiological functions. They digest proteins in our food, determine the activity of hormones, induce cell death and regulate blood clotting, for example. During viral infection, however, some proteases can switch sides and activate viral glycoproteins, allowing the entry of virions into new target cells and the spread of infection. To reduce unwanted effects, multiple protease inhibitors regulate the proteolytic processing of self and non-self proteins. This review summarizes our current knowledge of endogenous protease inhibitors, which are known to limit viral replication by interfering with the proteolytic activation of viral glycoproteins. We describe the underlying molecular mechanisms and highlight the diverse strategies by which protease inhibitors reduce virion infectivity. We also provide examples of how viruses evade the restriction imposed by protease inhibitors. Finally, we briefly outline how cellular protease inhibitors can be modified and exploited for therapeutic purposes. In summary, this review aims to summarize our current understanding of cellular protease inhibitors as components of our immune response to a variety of viral pathogens.

## 1. Introduction: Proteolytic Maturation of Viral Glycoproteins

Enveloped viruses face a dilemma: on the one hand, newly produced virions need to contain viral glycoproteins that mediate the binding and subsequent entry of the virus into new target cells. On the other hand, viral glycoproteins can hinder efficient spread if they bind to receptors in or on the producer cell and result in unwanted superinfection of already infected cells. To overcome this challenge, viruses have evolved several different strategies. While some viruses downmodulate their entry receptors on infected cells, others prime and activate their glycoproteins at a later step in the viral replication cycle. In fact, many viral glycoproteins require one or more proteolytic maturation steps to render newly produced virions fully infectious.

**Figure 1:**
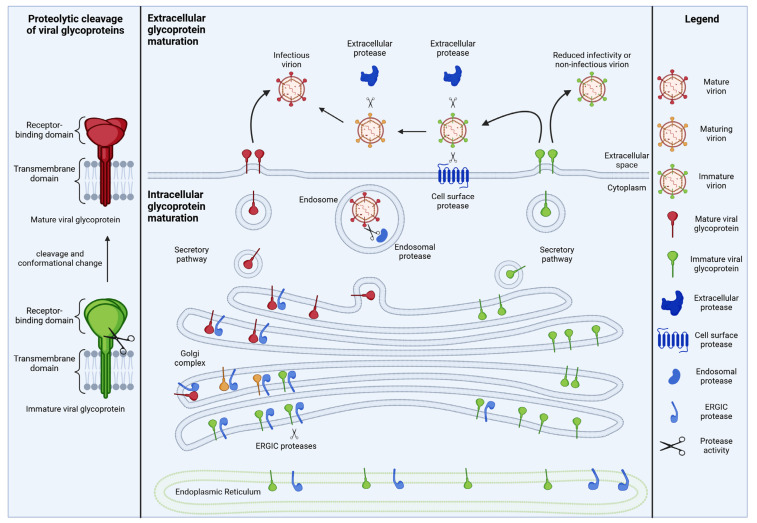
The maturation of viral glycoproteins. Many viral glycoproteins require one or more proteolytic activation steps separating the extracellular domain from the transmembrane domain and inducing a conformational change (**left**). These cleavage events are frequently mediated by cellular proteases and can occur in producer cells (e.g., in the ER or Golgi), in the extracellular space or at the plasma membrane (**right**).

Typically, the proteolytic cleavage of viral envelope glycoproteins separates the N-terminal extracellular part harboring the receptor-binding domain from the C-terminal part comprising the transmembrane domain and fusion peptide (Figure 1) [1]. While the mature glycoprotein fragments usually remain linked via disulfide bonds, the cleavage step induces conformational changes. These result in the liberation of functional domains such as the fusion peptide, thereby facilitating membrane fusion and entry into target cells [2,3,4]. Furthermore, cleavage-induced structural changes can affect the antigenicity of viral glycoproteins by exposing or masking epitopes [5,6]. Thus, a proteolytic activation of viral glycoproteins may also represent a mechanism allowing viruses to reduce their risk of detection by (neutralizing) antibodies.

Interestingly, the proteolytic cleavage of viral envelope proteins is usually mediated by cellular proteases that serve as virus dependency factors [1]. This reliance on host- rather than virus-encoded proteases allows viruses to keep their genome size to a minimum. However, this dependency may also come at a cost, since the activity of host proteases can be restricted to specific cell types, host species or time points. This may also explain why other proteolytic maturation steps during the viral replication cycle are mediated by viral proteases, at least in some virus species. For example, HIV-1 uses its own protease to cleave the viral Gag-Pol polyprotein into mature structural proteins and enzymes but uses a host protease to activate its envelope protein (Env) [7]. Similarly, the pp1a and pp1ab polyproteins of SARS-CoV-2 are processed by two viral proteases (PL^pro^ and M^pro^), whereas the Spike glycoprotein is activated by different cellular proteases [8,9]. 

In a prototypical maturation cascade, the viral glycoprotein enters the canonical secretory pathway and migrates through the ER and the Golgi to the cell surface where it can be released as a part of newly produced virions. Depending on the virus and its respective host protease, the proteolytic activation of viral glycoproteins can occur in different subcellular compartments (Figure 1). 

For example, the Env glycoprotein of the human immunodeficiency virus HIV harbors a polybasic cleavage site (RXK/RR) that is processed and activated in the Golgi apparatus of producer cells by the serine protease furin/PCSK3 or related proprotein convertases [10,11]. A similar intracellular furin-mediated priming step occurs at the S1/S2 site of SARS-CoV-2 Spike prior to the release of newly produced virions [12]. However, unlike HIV Env, SARS-CoV-2 Spike requires a second cleavage step at the so-called S2′ site for full activation [12,13]. S2′ cleavage can be mediated by the serine protease TMPRSS2 on the surface of SARS-CoV-2 target cells or by endosomal cathepsins [14]. Finally, some viruses can also be activated in the extracellular space by proteases that are not cell-associated. For example, the hemagglutinin (HA) protein of Influenza A viruses (IAVs) can be cleaved by a variety of extracellular proteases that are found in the respiratory tract (e.g., HAT and tryptase beta-2) and recognize monobasic target motifs [3]. 

Notably, the proteolytic activation of viral glycoproteins is also an important determinant of viral pathogenicity. Viruses that use ubiquitously expressed proteases such as furin may cause systemic infections, whereas viruses that rely on proteases with a limited expression pattern may also have a limited tissue tropism. For example, Newcastle disease viruses (NDVs) can be grouped into virulent and avirulent strains, which differ in the cleavage sites of their fusion (F) proteins. While the F proteins of virulent strains harbor prototypical PCSK cleavage sites (e.g., RQKR), their avirulent counterparts do not [15,16]. The acquisition of a polybasic PCSK cleavage site enables the intracellular activation of the F protein by ubiquitously expressed proteases such as furin and related PCSKs. In contrast, avirulent NDV strains depend on trypsin-like proteases found only in some tissues [15]. Similarly, highly pathogenic avian influenza viruses (HPAIVs) harbor a polybasic PCSK cleavage site in their HA proteins, enabling activation in a broader range of tissues compared to low pathogenic influenza viruses that harbor monobasic cleavage sites [17]. Another interesting example are feline coronaviruses (FCoVs). While some FCoVs cause severe peritonitis and are able to infect myeloid cells, others cause only mild enteritis and fail to infect macrophages or monocytes. These differences in tropism have been mapped to the C-terminal S2 domain of Spike. Inhibitor studies revealed that enteric viruses depend on both cathepsin B and L, whereas cathepsin L is dispensable for FCoVs causing peritonitis [18]. Together, these examples illustrate that mutations outside the receptor-binding domain may facilitate viral entry into specific target cells by altering host protease dependency. 

At first glance, the exploitation of host proteases looks like an easy game for the virus, where the host can only stand by and watch. Getting rid of virus-activating proteases is not an option for the host, as they fulfil important physiological functions and also activate a variety of cellular proteins. However, cells have evolved regulatory mechanisms to limit the proteolytic activation of viral proteins, while still allowing for sufficient processing of cellular proteins. In recent years, an increasing number of cellular proteins inhibiting protease activity have been described. These protease inhibitors can help our immune system to reduce virion infectivity by interfering with proteolytic viral glycoprotein maturation. Some of them are induced by IFNs or enhance IFN responses on their own [19,20,21,22], further highlighting a specific role in innate immunity. Antiviral protease inhibitors act by several different mechanisms: Some are secreted and inhibit proteases in the extracellular space, while others prevent viral protein cleavage intracellularly. Some irreversibly block the catalytic site, while others sequester their target proteases to specific subcellular compartments. In this review, we will summarize our current knowledge of cellular protease inhibitors that are known or suspected to restrict viral spread by preventing the maturation of viral glycoproteins. Where known, we will also describe their mode of action and potential viral escape mechanisms. 

## 2. Endogenous Inhibitors of Viral Glycoprotein Cleavage

### 2.1. Serine Protease Inhibitors (Serpins)

Serpins are the largest family of protease inhibitors, with 37 members in humans [23]. They can be found in all kingdoms of life [24]. Some viruses even encode members of the serpin superfamily [24,25]. The first serpins to be isolated were alpha1-anti-trypsin (=Serpin A1) and anti-thrombin (=Serpin C1). Together with ovalbumin, they were grouped into a new protein superfamily in 1980 [26], and the term ‘serpin’ was coined as an abbreviation of serine protease inhibitor. Indeed, many serpins inhibit serine proteases such as thrombin or human neutrophil elastase. However, some of them are cross-class inhibitors, inactivating cysteine proteases such as caspases, for example [27,28]. Many serpins inhibit their target proteases via an irreversible, non-competitive suicide mode of action. The exact mode of inhibition could be resolved with the description of serpin crystal structures [29]. Like a physiological protease target, serpins are bound by the active catalytic site and induce the formation of an acyl-enzyme intermediate. However, this intermediate is not resolved via hydrolysis but rather induces a rapid conformational change of serpin, resulting in the formation of inactive covalently linked serpin–protease heterodimers [30,31]. 

**Figure 2:**
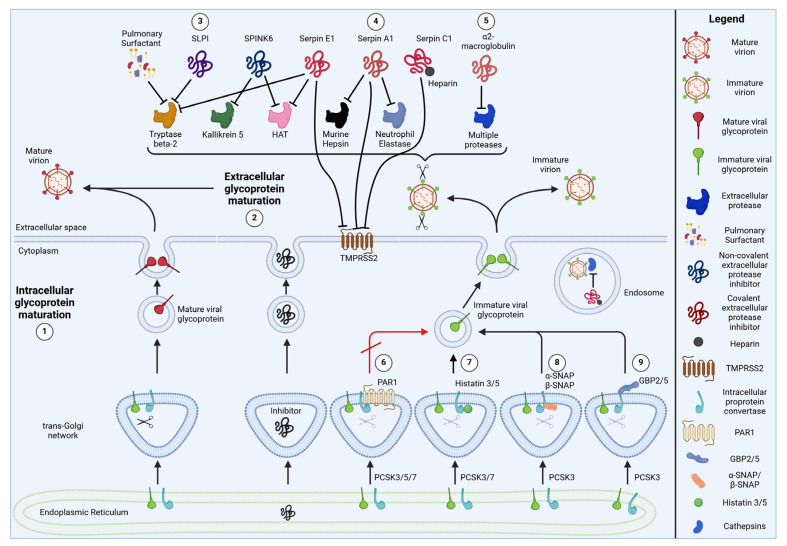
Cellular protease inhibitors suppressing viral glycoprotein maturation. Many viral glycoproteins are activated through proteolytic cleavage, mediated by cellular proteases. Proteolytic maturation can occur (1) intracellularly and/or (2) extracellularly, depending on the viral pathogen. To combat viral glycoprotein maturation, host cells have developed numerous strategies. Often, cells release inhibitors that target viral dependency factors extracellularly. For example, non-covalent extracellular protease inhibition has been described for SLPI and SPINK6 but also for pulmonary surfactant (3). In contrast, Serpin A1, C1 and E1 most likely inhibit their extracellular targets via a covalent mode of action (4). In this process, Serpin C1 relies on its allosteric activator Heparin to become fully active. The extracellular α2-macroglobulin, on the other hand, harbors typical protease target sequences as a bait. Upon proteolytic cleavage, a major conformational change occurs, trapping the target protease in a Venus flytrap-like mechanism (5). Other protease inhibitors target glycoprotein maturation intracellularly. For example, the G protein-coupled receptor PAR1 inhibits furin, PCSK5 and PCSK7 by forming inactive complexes with these proteases (6). As a result, the complexes are sequestered in the trans-Golgi network. In contrast, histatins 3 and 5 have been proposed to act as reversible and competitive inhibitors of furin and PCSK7 (7). α-SNAP inhibits furin by binding to its regulatory P domain (8). As a result, furin is still able to bind to its substrate but fails to efficiently process viral glycoproteins. Its homologue β-SNAP is likely to act via the same mechanism. Guanylate-binding proteins 2 and 5 restrict the proteolytic activity of furin (and potentially PCSK8) by binding to the cytoplasmic tails of these proteases (9).

Given their broad spectrum of target proteases, it is not surprising that serpins are involved in a variety of physiological and pathophysiological processes, including coagulation, inflammation and tissue remodeling [32]. For example, Serpin A1 deficiency results in severe emphysema and cirrhosis due to the hyperactivity of lung and liver proteases [33]. Similarly, Serpin C1 deficiency results in aberrant activation of the proteolytic coagulation cascade, and affected patients suffer from increased risk of thrombosis [34]. Most serpin studies initially focused on their interplay with cellular protein processing. It took until 2015 to discover that serpins also interfere with viral glycoprotein cleavage and can be important determinants of virion infectivity [35] (Figure 2). As outlined in more detail below, our knowledge of the antiviral activity of serpins was based on a few studies only [35,36,37] until serpin research was significantly boosted by the SARS-CoV-2 pandemic.

#### 2.1.1. Serpin E1/Plasminogen Activator Inhibitor 1 (PAI-1)

In 2015, an ISG screening identified Serpin E1 as an endogenous inhibitor of Influenza A virus replication [35]. The authors showed that Serpin E1 suppresses the activity of at least three different host proteases known to activate the viral HA protein: Human airway trypsin (HAT), tryptase beta-2 and TMPRSS2 (Table 1). In contrast, the study did not confirm a previously reported interaction of Serpin E1 with furin [38]. Consistent with these findings, Serpin E1 inhibited TMPRSS2-mediated cleavage of the HA of A/Puerto Rico/8/34 (H1) and A/Japan/305/57 (H2). In contrast, the cleavage of A/Vietnam/1203/2004 (H5) mediated by intracellular proteases such as furin was not affected. Furthermore, Serpin E1 suppressed the spread of IAV WSN/33 and Sendai virus but not furin-dependent human parainfluenzavirus 3 (HPIV3) in cell culture. Notably, a naturally occurring polymorphism (A43T) of Serpin E1, resulting in its partial intracellular retention, increased the spread of IAV WSN/33. Finally, Serpin E1^−/−^ mice showed increased IAV titers and pathology compared to wild-type controls. Further evidence for the role of Serpin E1 in antiviral immunity comes from its expression pattern. While several cell types constitutively express Serpin E1, its expression can be further upregulated by IFNs and TNFα [21] or viral infection [35]. 

More recently, Serpin E1 (along with Serpin A1, E2 and F1) was also identified in a screening for host factors that determine the susceptibility of primary lung cells to SARS-CoV-2 infection ex vivo [39]. Surface plasmon resonance assays confirmed that Serpin E1 shows a high-affinity interaction with TMPRSS2. As expected, TMPRSS2-mediated processing of SARS-CoV-2 Spike was inhibited, and SARS-CoV-2 replicated less efficiently in the presence of recombinant Serpin E1 [39]. The expression of Serpin E2, a closely related paralog of Serpin E1 [56], was also associated with reduced susceptibility to SARS-CoV-2 infection [39]. However, its effect on the proteolytic processing of SARS-CoV-2 Spike and other viral glycoproteins remains to be determined. While both Serpin E1 and Serpin E2 are expressed in the respiratory tract, single-cell sequencing revealed that they are primarily produced by basal cells, also during SARS-CoV-2 infection [39]. Thus, it remains unclear whether/how these serpins reach TMPRSS2 at the apical surface, the natural site of SARS-CoV-2 infection.

#### 2.1.2. Serpin A1/Alpha1-Anti-Trypsin

In contrast to Serpin E1 and E2, Serpin A1 shows a broader expression pattern in the respiratory tract, being expressed not only in basal cells but also in ciliated cells and goblet cells [39]. Not surprisingly, Serpin A1 was also found in the apical secretions of the air–liquid interface (ALI) cultures of human bronchial epithelial cells (HBECs) [39].

In response to the COVID-19 pandemic, several research groups independently showed that Serpin A1 reduces TMPRSS2-mediated SARS-CoV-2 infectivity [39,40,41]. For example, Wettstein and colleagues identified Serpin A1 as an inhibitor of SARS-CoV-2 by screening a peptide/protein library of pooled bronchoalveolar lavage (BAL) samples [41]. Using surface plasmon resonance, they demonstrated a direct binding of Serpin A1 to TMPRSS2. They also showed that TMPRSS2 activity and SARS-CoV-2 replication are inhibited in the presence of this protease inhibitor [41]. Similarly, Azouz and colleagues showed that Serpin A1 binds to the extracellular domain of TMPRSS2, inhibits its proteolytic activity and reduces SARS-CoV-2 infectivity [40]. These findings were confirmed by Rosendal et al., who directly demonstrated that the cleavage of SARS-CoV-2 Spike is reduced in the presence of Serpin A1 [39]. Structural modeling suggests that Serpin A1 covalently binds to TMPRSS2, resulting in proteolytically inactive heterodimers [40,41].

Importantly, Serpin A1 may protect us from severe COVID-19 by several independent mechanisms. For example, in addition to reducing Spike cleavage, Serpin A1 can inhibit thrombus formation [57] or prevent severe acute lung injury mediated by neutrophil elastase [58]. Intriguingly, Serpin A1-mediated inhibition of neutrophil elastase may potentially also directly affect Spike cleavage: SARS-CoV-2 variants carrying the D614G mutation in Spike harbor a predicted elastase cleavage site that has been proposed to increase viral fitness in Serpin A1-deficient populations [59]. 

Nevertheless, the protective role of Serpin A1 during SARS-CoV-2 infection is still controversial. While some studies suggest that Serpin A1 deficiency increases the risk of severe COVID-19 [60,61,62], others found no evidence for such an association [63,64]. Given that Serpin A1 is an acute phase protein [65], it is not surprising that Serpin A1 levels are elevated in COVID-19 patients. However, Serpin A1 levels positively correlate with COVID-19 severity [40,66], suggesting that its protective effects are rather limited in vivo.

While the interplay of Serpin A1 and SARS-CoV-2/COVID-19 has been studied in detail, the effects of Serpin A1 on other viral pathogens remain less well understood. In 2015, before the SARS-CoV-2 pandemic, Esumi and colleagues identified TMPRSS2 as a dependency factor of Hepatitis C virus (HCV) and demonstrated that Serpin A1 reduced HCV infectivity [36]. However, the exact target(s) of TMPRSS2-mediated cleavage during HCV replication remain to be determined.

Similarly, Serpin A1 reduced the replication of IAV Aichi/H3N2/PR8 in *Tmprss2-Tmprss4*-deficient mice [37]. The authors showed that Serpin A1 potently inhibits the proteolytic activity of murine Hepsin, which activates H3 in vitro. In contrast, the human Hepsin ortholog does not activate IAV HA [37,67]. Thus, the exact role of Serpin A1 during IAV replication in humans and the relevant target proteases have remained unclear. 

Finally, Serpin A1 was shown to inhibit the replication of HIV-1. People living with HIV-1 are characterized by lower serum levels of Serpin A1 compared to uninfected controls [68], and HIV-1 replicates more efficiently in peripheral blood cells from Serpin A1-deficient individuals [69]. Recombinant Serpin A1 reduced viral capsid protein (p24) production when added to producer cells and reporter gene expression when added during the infection of new target cells [69]. These antiviral effects are most likely independent of altered HIV-1 Env maturation that already occurs in producer cells but would not affect p24 production. Indeed, mechanistic analyses revealed that Serpin A1 inhibits the activation of NF-κB and reduces the degradation of its inhibitor IκB [69,70]. Reduced NF-κB-driven activation of the viral LTR promoter may explain the inhibition of p24 production, reporter gene expression and viral spread. Finally, Serpin A1 has also been proposed to directly interact with the HIV-1 Env subunit gp41, thereby blocking viral entry [71]. These findings illustrate that serpins may also interfere with viral replication without affecting the proteolytic maturation of viral glycoproteins. 

#### 2.1.3. Serpin C1/Anti-Thrombin III

Like Serpin E1 and A1, Serpin C1/anti-thrombin III is also involved in the regulation of the coagulation cascade [72]. In addition to its eponymous ability to inhibit thrombin (FIIa), it also suppresses the activity of the coagulation factors FIXa, FXa and FXIa [72]. When Rosendal and colleagues characterized the effects of Serpin A1 and E1 on SARS-CoV-2, they included Serpin C1 as a control since its anticoagulant effect was proposed to support the therapy of COVID-19 patients. Interestingly, Serpin C1 also efficiently bound to TMPRSS2 and inhibited TMPRSS2-mediated cleavage of SARS-CoV-2 Spike. Serpin C1 suppressed SARS-CoV-2 replication in HBEC ALI cultures even more efficiently than Serpin A1 or E1 [39]. These findings were extended by Wettstein and colleagues, who demonstrated that Serpin C1 reduced not only the infectivity of viral particles carrying SARS-CoV-2 Spike but also virions carrying the Spike proteins of SARS-CoV, MERS-CoV and hCoV-229E [42]. As expected, virions pseudotyped with the protease-independent glycoprotein G of vesicular stomatitis virus (VSV) were not restricted. Protease reporter assays revealed that Serpin C1 also suppresses the enzymatic activity of cathepsin L and to a much lesser extent cathepsin B [42], both of which can also cleave the S2′ site of SARS-CoV-2 Spike [14]. In contrast, furin activity was not inhibited [42]. 

In the prevention and treatment of thrombosis, the inhibitory activity of endogenous Serpin C1 is routinely enhanced by heparin or heparin-derived compounds such as Fondaparinux. When bound to Serpin C1, these compounds increase the flexibility of its reactive center loop (RCL), thereby enhancing its protease inhibitory activity. In line with this, heparin and Fondaparinux both boosted the anti-SARS-CoV-2 activity of Serpin C1 in human lung cells in vitro [42]. This finding demonstrates that enhancers of endogenous protease inhibitors may not only allow us to treat coagulation disorders but could potentially also be repurposed for the treatment of viral infections.

### 2.2. α2-Macroglobulin

α2-macroglobulin is one of the largest protease inhibitors, forming 720 kDa homotetramers [73]. It can be found in plasma, saliva and secretions of the respiratory tract [74,75], where it inhibits proteases from all classes, including serine, cysteine and metalloproteinases [76]. Due to its broad target spectrum, it is often referred to as a panprotease inhibitor. α2-macroglobulin harbors a bait region that acts as an activation domain and comprises target motifs for many proteases [76]. The proteolytic cleavage of the bait region induces a major conformational change of the inhibitor. In a Venus flytrap-like mechanism, α2-macroglobulin collapses over its target protease, thereby sterically shielding its catalytic site [76,77]. At the same time, a C-terminal receptor-binding domain is exposed, enabling binding to clearance receptors and the removal of the inactivated protease from the bloodstream [78]. 

α2-macroglobulin has been shown to be a major determinant of the Influenza A virus’s neutralizing activity of horse, pig and guinea pig sera [79,80,81]. This antiviral activity was ascribed to the oligosaccharide moieties of α2-macroglobulin, which compete with cellular entry receptors for hemagglutinin binding [79,80,81]. More recently, human salivary α2-macroglobulin was also shown to inhibit Influenza A virus infection [43]. The authors hypothesize that this protective effect is not only the result of receptor competition but also involves an inhibition of host proteases activating the viral hemagglutinin [43]. However, direct evidence for reduced IAV HA processing in the presence of α2-macroglobulin is missing. Furthermore, the neutralizing activity of α2-macroglobulin correlated with IAV receptor usage [80], suggesting that receptor competition may be the main mode of action.

Given its broad target spectrum, it is not surprising that α2-macroglobulin also interferes with the activity of viral proteases. For example, HIV protease also cleaves the bait region and is subsequently entrapped and inactivated by the inhibitor [82,83,84]. However, the physiological relevance of this inhibition remains unclear since HIV protease is usually not released from cells or virions and may not encounter α2-macroglobulin in vivo.

### 2.3. Protease-Activated Receptor 1 (PAR1)

PAR1 belongs to the family of protease-activated receptors. These G protein-coupled receptors regulate coagulation cascades and pro-inflammatory signaling pathways upon proteolytic activation [85]. For example, PAR1 serves as a target for thrombin, which cleaves PAR1 N-terminally [86]. The N-terminal PAR1 fragment remains bound as a tethered ligand and results in the irreversible activation of PAR1. 

In 2013, Aerts et al. showed that PAR1 overexpression reduced the proteolytic cleavage of the human metapneumovirus F protein [44]. Interestingly, however, stimulation with the PAR1 agonist TFLLR-NH_2_ rescued F protein cleavage in transfected HEK293T cells and increased furin expression in the lungs of mice infected with human metapneumovirus (hMPV). Consistent with these findings, intranasal administration of the PAR1 agonist also increased the mortality of hMPV-infected mice [44]. The authors hypothesize that unactivated PAR1 at the plasma membrane may inhibit the cleavage of the F protein. However, upon activation, PAR1 is rapidly internalized and degraded [87], which may explain the increased F protein cleavage in the presence of the PAR1 agonist.

In line with the findings of Aerts and colleagues, PAR1 was later shown to inhibit the proteolytic cleavage of HIV-1 Env and infectious virus yield in transfected HEK293T cells [45]. This antiviral activity of PAR1 was attributed to an inhibition of furin, PCSK5 and—to a lesser extent—PCSK7 [45]. In contrast to PAR1, its paralog PAR2 fails to inhibit furin-mediated protein cleavage. Mechanistic analyses revealed that PAR1 forms inactive complexes with furin that are trapped in the trans-Golgi network, a process that depends on the adapter protein PACS1. In contrast to membrane-bound furin and PCSK5/PC5B, soluble PCSK5/PC5A and PCSK6/PACE4 cleave PAR1 at an R_41_XXXXR_46_↓ motif [45]. This cleavage abrogates PAR1’s responsiveness to thrombin, which cleaves at R_41_↓. In line with a potential role in restricting viral replication, PAR1 expression is increased under pro-inflammatory conditions such as the brains of HIV-1-infected individuals suffering from HIV-associated neurocognitive disorders (HANDs) [45].

In addition to its ability to reduce virion infectivity by interfering with viral glycoprotein maturation, PAR1 may also affect viral disease progression via its ability to induce immune signaling. On the one hand, PAR1 may be protective and limit viral replication by inducing IFN-β expression [19]. On the other hand, however, PAR1 may also promote detrimental immune activation. For example, PAR1 was shown to promote deleterious pro-inflammatory responses and reduce the survival of Influenza A virus-infected mice [88]. Similarly, the inhibition of PAR1 reduced viral replication and inflammation in mice infected with RSV or hMPV [89]. Since increased thrombin activity may contribute to the pathogenesis of acute respiratory distress syndrome in SARS-CoV-2-infected individuals, the thrombin receptor PAR1 has also been proposed as a potential target for the therapy of COVID-19 [90]. Taken together, these studies illustrate that the roles of PAR1 in viral infection and disease are complex, involving direct effects on virion infectivity and indirect effects on immune activation. 

### 2.4. Histatins 3 and 5

Histatins are histidine-rich cationic proteins that are secreted by salivary glands [91]. They have been shown to exert anti-microbial activity, for example, by disrupting the membranes of fungal cells [91]. Furthermore, they inhibit bacterial and mammalian proteases, including viral dependency factors [46,92,93,94]. For example, histatin 5 and (to a lesser extent) histatin 3 suppress the activity of furin and PCSK7, while serving as substrates for PCSK1 [46]. Histatin 5 represents an N-terminal fragment of histatin 3. Interestingly, both histatins share some sequence similarity with the C-terminal halves of the inhibitory prodomains of furin and PCSK7 [46]. Whether this sequence similarity is involved in the inhibition of furin and PCSK7 remains to be determined. 

Of note, histatin 5 is able to restrict HIV-1 infection [47]. Thus, it may be tempting to speculate that histatins interfere with the proteolytic processing of HIV-1 Env and possibly other PCSK-dependent viral glycoproteins. However, in the HIV-1 study mentioned above, histatin 5 was co-incubated with infectious viral particles during the time point of infection. Since HIV-1 Env is usually proteolytically activated inside producer cells, these viral particles most likely harbored active Env proteins that had already been cleaved. Consequently, the observed reduction in HIV-1 infection in the presence of histatin 5 is most likely not the result of reduced PCSK-mediated Env processing [47]. Surprisingly, histatin 5 did not significantly affect infection with lentiviral particles that were pseudotyped with HIV-1 Env [47]. This finding suggests that Env is not the only determinant of histatin 5 sensitivity. In an earlier study, histatin 5 variants with increased antifungal activity had been generated [95]. Unexpectedly, these derivatives increased rather than decreased HIV-1 infection [47]. The reasons for these differences between wild-type and mutated histatin 5 have remained unclear. In summary, the interplay of histatins with HIV-1 infection are enigmatic, and PCSK-independent modes of action should be considered. 

### 2.5. Alpha- and Beta-Soluble N-Ethylmaleimide-Sensitive Factor Attachment Protein (α-SNAP, β-SNAP) 

SNAP proteins are primarily known for their role in vesicular membrane fusion. Here, they mediate vesicle fusion events by associating with proteins of the SNARE (SNAP receptor) complex and the ATPase NSF (N-ethylmaleimide-Sensitive Factor) [96]. More recently, α-SNAP was identified in a mass spectrometry-based screening for interaction partners of SARS-CoV-2 Spike [22]. Subsequent overexpression revealed impaired proteolytic cleavage of SARS-CoV-2 Spike in the presence of α-SNAP. This suppressive activity could be ascribed to a direct binding and inhibition of furin by α-SNAP in the ER and Golgi [22]. In addition, the proteolytic separation of the catalytic site of furin with the C-terminal transmembrane protein was less efficient in the presence of α-SNAP, suggesting that furin shedding is also impaired. Notably, α-SNAP does not block the catalytic site of furin but interacts with its P domain [22]. This regulatory domain determines the stability, pH optimum and Ca^2+^ dependence of furin and related proprotein convertases [97]. As a result, furin still binds to Spike in the presence of α-SNAP, but it fails to efficiently cleave and activate this viral protein. Further mechanistic analyses revealed that the NSF-interacting C-terminus is dispensable for the ability of α-SNAP to inhibit furin [22].

In line with its ability to target furin, α-SNAP also reduced the proteolytic cleavage of Ebola virus and Marburg virus Gp, as well as MERS-CoV Spike [22]. In contrast, furin-independent cleavage of the Spike protein of SARS-CoV was unaffected. Notably, type I and type III interferons significantly increased α-SNAP expression in HEK293, A549 and Calu-3 cells, further pointing towards the important role of this protein in antiviral immunity [22]. 

Humans encode three SNAP proteins: while α-SNAP and γ-SNAP are expressed in most cell types and tissues, β-SNAP expression is largely restricted to the brain [96]. Intriguingly, β-SNAP, which has high sequence similarity with α-SNAP, also inhibits SARS-CoV-2 Spike cleavage, whereas γ-SNAP shows no such antiviral effect [22]. Thus, at least two of the three human SNAP proteins may play a role beyond vesicle fusion and exert broad antiviral activity by inhibiting the maturation of a variety of furin-dependent viral glycoproteins.

### 2.6. Guanylate-Binding Proteins 2 and 5 (GBP2 and GBP5)

GBPs belong to the family of large IFN-inducible GTPases and protect us from viral, bacterial and parasitic infections via a number of different mechanisms [98]. In 2016, GBP5 was identified in a screening for antiretroviral host factors, where it reduced the infectivity of HIV-1 virions [99,100]. This antiretroviral activity was subsequently shown to be shared by its paralog GBP2, although to a slightly weaker extent [20]. Reduced HIV-1 infectivity could be ascribed to reduced proteolytic maturation of the viral Env protein [20]. In line with this, GBP2 and GBP5 both bind to the cytoplasmic tail of furin and inhibit its proteolytic activity [20]. Furthermore, the (auto)catalytic processing of furin and its shedding into the extracellular space are reduced in the presence of GBP2 or GBP5 [20]. Mutational analyses revealed that furin inhibition requires the presence of a C-terminal isoprenylation site and membrane anchor in GBP2 or GBP5, whereas their GTPase activity is dispensable [20,99]. Since many viral pathogens use furin for the maturation of their glycoproteins, it is not surprising that GBP2 and GBP5 exert a rather broad antiviral activity. In addition to HIV-1, GBP2 and GBP5 also restrict the replication of Zika virus, measles virus and IAV A/seal/Mass/1-SC35M, as well as the proteolytic maturation of murine leukemia virus (MLV) glycoprotein (GP) and IAV HA [20]. Furthermore, GBP2 and GBP5 reduce the infectivity of pseudovirions carrying the glycoproteins of Marburg virus and Rabies virus. In contrast, virions pseudotyped with VSV G, which does not require a furin-mediated activation step, are not restricted [20]. More recently, GBP2 and GBP5 have also been shown to reduce SARS-CoV-2 infectivity by inhibiting the proteolytic processing of Spike [48]. However, as described in more detail below, some SARS-CoV-2 isolates were resistant to GBP-mediated restriction [48].

Another virus that is restricted by GBP5 and uses furin for the cleavage of its glycoprotein is respiratory syncytial virus (RSV) [101]. As with the above viruses, the activity of GBP5 against RSV depends on the presence of the C-terminal isoprenylation anchor. However, GBP5-mediated restriction was attributed to reduced intracellular levels of the viral small hydrophobic (SH) protein [101]. Whether GBP5-mediated impairment of the cleavage of the RSV glycoprotein F also contributes to restriction has remained unclear. Interestingly, GBP2 and GBP5 also suppressed the maturation and fusogenic activity of an endogenous retroviral Env protein (HERV-K), suggesting that these furin inhibitors may have protected our ancestors from ancient retroviral infections [49]. 

Pseudotyping experiments revealed that the glycoprotein GP-C of Lassa virus (LasV) is also sensitive to GBP2- and GBP5-mediated restriction [20]. Intriguingly, LasV uses PCSK8/S1P instead of PCSK3/furin to activate its glycoprotein [102]. It is therefore tempting to speculate that GBPs may target not only furin but also other members of the PCSK family of proteases. 

### 2.7. Membrane-Associated RING-CH 8 (MARCH8)

In 2015, the Tokunaga laboratory identified the E3 ubiquitin ligase MARCH8 as an antiviral host factor that restricts the entry of virions carrying HIV-1 Env or VSV G [103]. This inhibitory activity was attributed to decreased levels of the viral glycoproteins at the plasma membrane, resulting in reduced virion incorporation and thus reduced infectivity of newly produced viral particles [103]. A systematic comparison of different MARCH proteins revealed that MARCH1 and MARCH2 share the antiviral activity of MARCH8 [104]. Subsequent mechanistic studies showed that MARCH8 targets VSV G and HIV-1 Env via two distinct mechanisms: while the inhibition of VSV G involves the ubiquitination of lysine residues in the C-terminal tail of this viral protein, the restriction of HIV-1 Env depends on a cytoplasmic sorting motif in MARCH8 [105]. In agreement with this, VSV G is degraded in the presence of MARCH8, while HIV-1 Env is sequestered in intracellular compartments [105]. More recently, MARCH8 has been shown to target an even wider range of viral glycoproteins, including Ebolavirus Gp, IAV H5N1 HA, SARS-CoV-2 Spike or Rabies virus G, for example [50,51,106]. Intriguingly, many of the viral MARCH8 targets can be cleaved and activated by furin, and Yu et al. observed reduced proteolytic processing of EBOV GP, HIV-1 Env and H5N1 HA in the presence of MARCH8 [51]. Similar inhibitory effects on viral glycoprotein cleavage were observed in two follow-up studies [50,52], suggesting that MARCH8 may suppress furin-mediated cleavage. Consistent with this hypothesis, MARCH8 co-immunoprecipitated with furin in interaction assays [51]. Notably, however, the original MARCH8 study by the Tokunaga laboratory did not observe altered HIV-1 Env cleavage in the presence of MARCH8 [103], and several furin-independent viral glycoproteins were also restricted [106]. Further studies are therefore required to investigate whether MARCH8 indeed inhibits the enzymatic activity of furin or whether altered glycoprotein processing is a consequence of altered intracellular trafficking. 

### 2.8. Serine Protease Inhibitor Kazal-Type 6 (SPINK6)

SPINK6 was first described as an inhibitor of kallikrein-related peptidases (KLKs) in skin [53]. Later, SPINK6 was also shown to be expressed in bronchial epithelial cells and further upregulated in airway organoids following infection with Influenza A virus [54]. Together with the observation that KLK5 and KLK12 can cleave certain Influenza A virus HA proteins and/or increase influenza virus infectivity [107,108], this raised the possibility that SPINK6 may interfere with the proteolytic activation of HA and protect us from Influenza A virus infection. Indeed, Wang and colleagues showed that SPINK6 limits IAV replication in human airway organoids and infected mice [54]. Further evidence for the antiviral activity of SPINK6 comes from GWAS data: individuals carrying a single-nucleotide polymorphism that increases SPINK6 expression have a reduced risk of H7N9 infection [54]. Mechanistic analyses revealed that SPINK6 inhibits not only KLKs but also the proteolytic activity of HAT/TMPRSS11D [54], one of the major serine proteases that activate HA in human airways [109]. In contrast, TMPRSS2 and furin are not inhibited by SPINK6 [109]. Taken together, these results identify SPINK6 as an infection-inducible protease inhibitor that restricts Influenza A virus replication in the respiratory tract and determines susceptibility to viral infection in humans. 

### 2.9. Secretory Leukocyte Protease Inhibitor (SLPI)

The 12 kDa protein SLPI, also known as Mucus Protease Inhibitor (MPI), is found in a variety of secretions, including those of the upper respiratory tract, reproductive mucosa and gastrointestinal tract [110]. SLPI inhibits a variety of proteases, including leukocyte elastase, cathepsin G and trypsin [111]. In 1997, Beppu and colleagues showed that tryptase beta-2 (=tryptase Clara) is also a target of SLPI [112]. As a result, tryptase beta-2-mediated cleavage of Sendai virus F and Influenza A virus HA was inhibited in the presence of SLPI [112]. Furthermore, SLPI reduces the infectivity of both viruses in a dose-dependent manner. As expected, SeV and IAV infectivity were not affected when the viruses were proteolytically activated prior to co-incubation with SLPI [112]. Notably, SLPI not only reduced virion infectivity in vitro but also suppressed IAV replication in rats when administered intranasally [112,113]. SLPI contains two whey acidic protein (WAP) domains [114]. The C-terminal WAP domain contains two residues (Leu72, Met73) that are essential for protease inhibition. In line with this, the C-terminal half of SLPI is sufficient to inhibit tryptase beta-2 activity, whereas the N-terminal half is not [112]. However, the C-terminal half is approximately one log less active compared to full-length SLPI, possibly because the N-terminus stabilizes the conformation of the C-terminus [115]. 

Apart from IAV and SeV, SLPI also inhibits the HIV-1 infection of primary monocytes and PBMCs [116,117]. Importantly, the SLPI concentrations required for efficient viral restriction in vivo are reached in saliva and colostrum [116,118], suggesting that SLPI contributes to the antiviral activity of these body fluids in a dose-dependent manner. Indeed, the partial depletion of SLPI decreased the anti-HIV-1 activity of saliva [116]. Although Jana and colleagues reported an induction of SLPI in oral epithelial cells exposed to (heat-inactivated) HIV-1 or recombinant gp120 [118], there is no evidence for differences in salivary SLPI concentrations between people living with HIV-1 and uninfected controls [118]. Mechanistic analyses suggest that SLPI does not directly target the virus, but rather a ~55 kDa protein on HIV-1 target cells. However, the anti-HIV-1 activity of SLPI is not dependent on its ability to inhibit proteases [119], and the C-terminal domain of SLPI is not sufficient to restrict HIV-1 [119]. Thus, unlike SLPI-mediated inhibition of IAV and SeV, the inhibition of HIV-1 most likely does not involve altered viral glycoprotein processing. Instead, SLPI appears to block HIV-1 infection after receptor binding, but before reverse transcription [119]. SLPI therefore is an interesting example of an antiviral host factor targeting diverse viruses via protease-dependent and -independent mechanisms. 

### 2.10. Pulmonary Surfactant

Surfactant is a mixture of lipids (primarily dipalmitoylphosphatidylcholine) and proteins (surfactant proteins A–D) that is produced by type II alveolar cells. By reducing surface tension, surfactant enables the lung to inflate more easily and prevents the collapse of alveoli. In addition to its role in lung inflation, surfactant exerts antiviral activity via at least two different mechanisms: First, surfactant proteins A and D (SP-A, SP-D) can opsonize viral pathogens via their lectin domains and mark them for elimination by phagocytes [120]. Second, surfactant inhibits the proteolytic activation of Influenza A virus HA and Sendai virus F proteins, most likely as a result of its ability to inhibit tryptase beta-2 (=tryptase Clara) in a non-competitive manner [55]. While individual surfactant components such as SP-A do not inhibit tryptase beta-2, a mixture of purified phosphatidylcholine, phosphatidylglycerol, phosphatidylserine and SP-A can recapitulate the inhibitory activity of endogenous surfactant [55]. Like SLPI, the amount of SP-A in bronchoalveolar lavage fluid can be elevated by ambroxol, possibly by increasing secretion rather than expression [113]. Although it also increased the secretion of trypsin-like proteases, ambroxol treatment prolonged the survival of Influenza A virus-infected mice [121]. Whether this protective effect is due to elevated surfactant and SLPI levels or a concomitant increase in IgA and IgG secretion remains to be determined [121]. Although the exact mechanism of inhibition remains unclear, the example of surfactant demonstrates that not only single proteins but also lipid–protein complexes can suppress viral glycoprotein maturation. 

## 3. Viral Evasion and Counteraction Strategies

### 3.1. Evasion 

Despite the presence of host factors limiting the availability of proteases for viral glycoprotein maturation, the pathogens listed above continue to spread efficiently in their infected hosts. This is in part due to viral evasion or backup strategies that ensure the generation of fully infectious virions.

#### 3.1.1. Increased Ratio of Protease to Protease Inhibitor 

**Figure 3:**
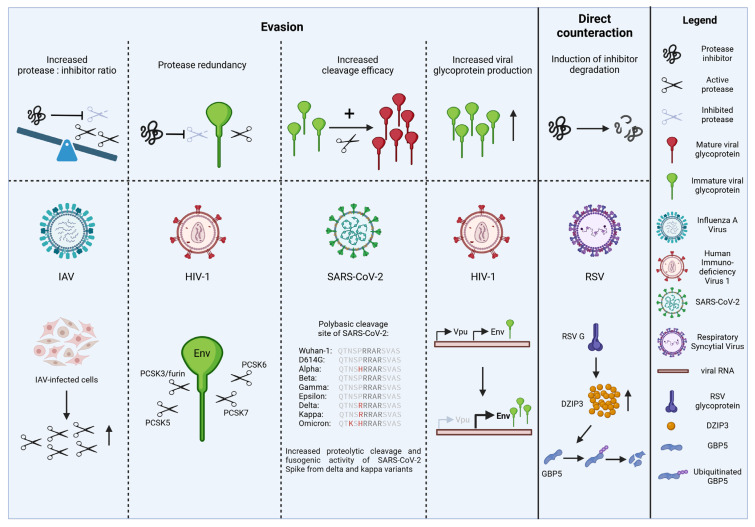
Viral evasion and counteraction mechanisms. Many viruses have evolved evasion or counteraction mechanisms that ensure the generation of fully infectious virions even in the presence of cellular protease inhibitors. For example, some viruses enhance the expression of the host protease(s) that activate their glycoproteins and/or decrease the levels of the respective inhibitors. Other viruses take advantage of several redundant proteases to ensure efficient cleavage or mitigate the effects of protease inhibitors by optimizing their cleavage sites or by increasing the amounts of virion-associated glycoproteins. More direct counteraction mechanisms include the degradation of protease inhibitors. The general principles of evasion and counteraction are illustrated on top; specific examples for individual viruses are shown at the bottom and described in Section 3.1 and Section 3.2.

For instance, many viruses induce the expression of proteases in infected cells, thereby shifting the protease vs. protease inhibitor balance in favor of the protease that activates the viral glycoprotein (Figure 3). A well-described example is the induction of HA-cleaving proteases upon IAV infection [17,54]. Similarly, Sendai virus and hMPV infection increase the expression of tryptase beta-2 [122] and furin [44], respectively. Conversely, some viruses reduce the production of protease inhibitors. A rather drastic example is the infection of salivary glands with SARS-CoV-2, which leads to their atrophy, fibrosis or ductal rupture and thus to a reduced release of the protease inhibitor histatin-5 [123]. 

#### 3.1.2. Redundant Use of Multiple Proteases

Another evasion strategy is the use of multiple (redundant) host proteases for the cleavage-mediated activation step. If the catalytic activity of one protease is inhibited, a second protease can compensate for it and still allow the generation of fully infectious virions. For example, the S2′ site of SARS-CoV-2 Spike cannot only be cleaved by TMPRSS2 at the plasma membrane but also by endosomal proteases such as cathepsin L or cathepsin B [14]. Thus, TMPRSS2-independent entry via endosomes may represent a backup strategy and ensure viral spread in the presence of endogenous TMPRSS2 inhibitors such as Serpin A1. Similarly, neutrophil elastase was shown to cleave SARS-CoV-2 Spike-derived peptides immediately downstream of the polybasic furin cleavage site [124]. Furthermore, the D614G mutation in Spike was suggested to result in the emergence of an additional elastase cleavage site upstream of the furin cleavage motif [59]. One interesting observation in this regard is that the D614G change allows the alpha and delta variants to escape GBP2/5-mediated restriction [48]. However, the exact role of neutrophil elastase in SARS-CoV-2 Spike maturation and a possible compensation of other Spike-priming proteases such as furin by neutrophil elastase remain to be determined.

Another example of protease redundancy are the polybasic cleavage sites of SARS-CoV-2 Spike, HIV-1 Env and other viral glycoproteins themselves. While these polybasic cleavage sites are often referred to as furin cleavage sites, related proprotein convertases recognize identical or similar target sequences [125,126]. For example, HIV-1 is not only prone to cleavage by furin/PCSK3 but also by PCSK5, 6 and 7 [10,11]. Consequently, host protease inhibitors targeting individual PCSK protein family members may not fully abrogate Env processing.

#### 3.1.3. Increased Cleavage Efficacy

If the amount of enzymatically active proteases is limited, the optimization of the proteolytic cleavage site may also allow viruses to mitigate the effects of protease inhibitors. During the SARS-CoV-2 pandemic, for example, several mutations in and around the polybasic cleavage site of Spike emerged. One notable example is the P681R change directly upstream of the polybasic S1/S2 site RRXR (positions 682–685) in the delta and kappa variants of SARS-CoV-2. This additional basic residue significantly increases furin-mediated cleavage and fusogenic activity of Spike [127] and may therefore confer a fitness advantage in the presence of cellular furin inhibitors.

#### 3.1.4. Increased Viral Glycoprotein Production

A more indirect escape mechanism is to increase virion infectivity via protease-independent mechanisms. For example, HIV-1 isolates with naturally occurring premature stop codons in Vpu are characterized by an increased expression of Env [20], since both viral proteins are translated from the same bicistronic RNA. As a result, budding virions incorporate more Env and are more infectious and less sensitive to GBP2/5-mediated restriction [20]. It is tempting to speculate that similar mechanisms may also allow other virions to reduce the restriction imposed by cellular protease inhibitors.

### 3.2. Direct Counteraction

In some cases, viruses do not evade or circumvent restriction but instead directly counteract an antiviral protein. The evolution of viral proteins that antagonize cellular antiviral factors illustrates the enormous selection pressure exerted by these host proteins. Direct counteraction strategies can include the degradation, inactivation or relocalization of antiviral host factors. 

For the protease inhibitors described above, however, only a limited number of direct viral counteraction mechanisms have been proposed to date. For example, RSV infection decreases GBP5 protein levels via its glycoprotein G [101]. More specifically, G increases the expression of the E3 ligase DZIP3 that induces K48 (but not K63) ubiquitination and subsequent proteasomal degradation of GBP5 [101]. ChIP revealed that G-mediated induction of DZIP3 coincides with increased H3K4 methylation of the respective gene locus. Strictly speaking, however, RSV G-mediated degradation of GBP5 is not a direct counteraction mechanism since it does not directly target the furin inhibitor but rather indirectly reduces its half-life [101]. 

## 4. Conclusions and Future Directions

Given the large number of proteases in the human body and their critical role in a plethora of physiological pathways, it is not surprising that their activity is tightly regulated by cellular protease inhibitors. Without these control mechanisms, aberrant protease activity can lead to serious disorders such as thrombosis. As a positive side effect, protease inhibitors also protect us from viral pathogens that exploit cellular proteases for their own purposes, primarily to activate their envelope glycoproteins. Thus, many protease inhibitors have also become important components of the antiviral immune response.

Still, our knowledge of the relative importance of individual host proteases for viral replication is limited, and only a few pairs of proteases and protease inhibitors have been characterized in detail in the context of viral glycoprotein maturation. A preprint describing the in silico modeling of inhibitor–protease interactions suggests that additional inhibitors of viral dependency factors such as furin or TMPRSS2 remain to be discovered [128]. The family of serpins, in particular, could still hold many discoveries of novel antiviral factors. Notably, most studies have also focused on only a few selected viral pathogens, in particular IAV and HIV-1 (Table 1). More recently, however, the emergence of SARS-CoV-2 has greatly stimulated research in this field, especially since the polybasic cleavage site in its Spike protein distinguishes SARS-CoV-2 from closely related sarbecoviruses [129].

Nevertheless, systematic analyses to identify those host proteases and potential inhibitors that are key determinants of viral particle maturation, virion infectivity and viral spread in vivo are often lacking. Systematic screenings and comparisons are hampered by several factors: (1) Viral glycoproteins can often be cleaved by multiple proteases, which in turn might be blocked by multiple inhibitors. Thus, the experimental depletion of individual proteases or their inhibitors may be compensated by redundant proteins masking potential effects. (2) In many cases, the inhibition of host proteases in cell cultures will affect not only viral protein maturation but also the processing of cellular protease substrates. Thus, some of the observed effects on viral replication may be indirect and not necessarily involve altered glycoprotein activation. (3) Similarly, in vivo data (e.g., from genome-wide association studies) may allow for the identification of proteases and their inhibitors involved in viral infection and disease but may not necessarily indicate whether altered viral glycoprotein maturation is the main underlying mechanism. (4) In vitro experiments using recombinant proteases, inhibitors and viral protein substrates allow us to control for potential cellular confounders. However, protease activity and inhibitor binding also strongly depend on factors such as pH, ion concentrations or the presence of co-activators. Thus, in vitro experiments may allow for a direct comparison of several proteases and their inhibitors but may not necessarily reflect the in vivo situation. In most cases, a combination of in vivo data and ex vivo or in vitro experiments will be required to fully elucidate the interplay of cellular protease inhibitors with their targets and their effect on viral protein maturation.

Not surprisingly, cellular proteases that serve as virus dependency factors also represent an attractive target for therapeutic intervention. For example, the TMPRSS2 inhibitors camostat and nafamostat had been approved for the treatment of pancreatitis in Japan and were repurposed for a potential treatment of COVID-19 since they efficiently block SARS-CoV-2 Spike cleavage in vitro [130]. Similarly, several cathepsin inhibitors have been tested for the treatment of COVID-19 [131,132]. 

Apart from the development of synthetic protease inhibitors (e.g., small molecules, peptide mimetics, nanobodies), endogenous protease inhibitors can also be modified and/or targeted for therapeutic approaches. For example, the induction of a protease inhibitor (e.g., via cytokines or CRISPRa) has the potential to increase the inhibitor-to-protease ratio, thereby preventing efficient viral glycoprotein activation. Apart from inducing inhibitor expression, the subcellular relocalization of an endogenous protease inhibitor also has the potential to restrict viral spread. An interesting example in this context is Serpin B8. This serpin has been shown to inhibit furin in vitro [133], but it does not prevent furin-mediated HIV-1 Env processing inside cells since it localizes to the cytosol rather than to the Env- and furin-containing secretory pathway [134]. In line with this, the addition of an N-terminal signal peptide relocalized Serpin B8 to the secretory compartment and enabled it to reduce HIV-1 particle infectivity [134]. While this is an interesting example of how a simple relocalization step can render a cellular protease inhibitor antivirally active, a direct therapeutic application using this approach is not in sight. Finally, several studies have modified cellular protease inhibitors to improve or change their affinity to and selectivity for specific substrates. Well-characterized examples are serpins harboring mutations in their reactive center loops (RCLs). One of the first serpin derivatives was alpha-1 anti-trypsin Portland variant (alpha 1-PDX), a variant of Serpin A1/alpha-1-anti-trypsin, which harbors a furin target sequence in its RCL [135]. While the mutations confer furin inhibitory activity to this serpin, its ability to inhibit thrombin is lost [135]. Consistent with potent furin inhibition, alpha 1-PDX reduced the proteolytic processing and fusogenic activity of HIV-1 Env and measles virus F protein [135,136] and suppressed HIV-1 replication in T cells [137]. Follow-up studies introduced additional mutations that increased the specificity of alpha 1-PDX for furin/PCSK3 [138,139] or changed it to S1P/PCSK8 [140]. As expected, the latter abolished PCSK8-mediated cleavage of Lassavirus GP-C and restricted Lassavirus replication [140]. Another interesting approach is the modification of the bait region of alpha-2 macroglobulin. For example, the replacement of the natural bait region by the cleavage site of tobacco etch virus (TEV) renders alpha-2 macroglobulin active against TEV protease [141].

Taken together, these examples illustrate how the study of cellular protease inhibitors can improve our understanding of innate antiviral immune responses and help to uncover viral dependencies that could be targeted for the prevention and/or therapy of viral diseases. In addition, a better understanding of the regulatory network of protease inhibitors and their targets will also be of interest beyond virology, as proteases such as furin also play key roles in a variety of physiological processes.

## Figures and Tables

**Table 1:** Cellular protease inhibitors suppressing proteolytic maturation of viral glycoproteins. Question marks (?) indicate hypotheses that are controversial or still need to be tested.

Cellular Protease Inhibitor	Target Proteases	Modes of Inhibition	Target Viruses (Viral Glycoprotein)	References
Serpin E1	HAT Tryptase beta-2 TMPRSS2	The formation of covalently linked inactive serpin-protease dimers	Influenza A virus (HA) Sendai virus (F) SARS-CoV-2 (Spike)	[35,39]
Serpin A1	TMPRSS2 Neutrophil elastase Murine Hepsin	Most likely the formation of covalently linked inactive serpin–protease dimers	SARS-CoV-2 (Spike) Influenza A virus (HA) Hepatitis C virus (unknown)	[36,37,39,40,41]
Serpin C1	TMPRSS2 Cathepsin L	Most likely the formation of covalently linked inactive serpin–protease dimers	SARS-CoV-2 (Spike) MERS-CoV (Spike) SARS-CoV (Spike) hCoV-229E (Spike)	[39,42]
α2-macroglobulin	Many	The cleavage of a bait region induces a conformational change in the inhibitor; in a Venus flytrap-like mechanism, the inhibitor collapses and masks the active site of the protease	Influenza A virus (HA) (?)	[43]
PAR1	PCSK3/furin PCSK5/PC5B PCSK7/PC7	The sequestration of inactive protease–PAR1 complexes in the trans-Golgi network; PACS1-mediated sequestration	HIV-1 (Env) hMPV (F)	[44,45]
Histatins 3 and 5	PCSK3/furin PCSK7/PC7	Reversible and competitive inhibition; sequence similarity with inhibitory PCSK prodomains (?)	HIV (Env) (?)	[46,47]
α-SNAP β-SNAP	PCSK3/furin	Interaction with the P domain of furin; substrate binding not affected	SARS-CoV-2 (Spike) MERS-CoV (Spike) Ebola virus (Gp) Marburg virus (Gp)	[22]
GBP2/GBP5	PCSK3/furin PCSK8/S1P (?)	The binding of the C-terminal cytosolic domain of furin; the inhibition of furin shedding; inhibition requires the isoprenylation of GBP2/GBP5 but not their GTPase activity; reduced shedding of furin	HIV-1 (Env) MLV (Env) HERV-K (Env) SARS-CoV-2 (Spike) Influenza A virus (HA)	[20,48,49]
MARCH8	PCSK3/furin (?)	Interaction with furin; the sequestration of furin/glycoprotein complexes in the Golgi	Ebola virus (Gp) Influenza A virus (HA) HIV-1 (Env)	[50,51,52]
SPINK6	HAT KLK5	Unknown	Influenza A virus (HA)	[53,54]
SLPI	Tryptase beta-2	Inhibition requires the C-terminal domain of SLPI (Leu72/Met73)	Sendai virus (F) Influenza A virus (HA)	
Surfactant	Tryptase beta-2	Non-competitive inhibition; multiple surfactant components required	Sendai virus Influenza A virus	[55]

## Data Availability

Not applicable.

## Abbreviations

A549, Adenocarcinomic human alveolar basal epithelial cells; ALI, Air-Liquid Interface; ATPase NSF, Adenosine 5′-TriPhosphatase N-ethylmaleimide-Sensitive Factor or fusion protein; BAL, Bronchoalveolar Lavage; Calu-3, Human Lung Adenocarcinoma Cells; COPD, Chronic Obstructive Pulmonary Disease; COVID-19, Coronavirus Disease 2019; CRISPRa, Clustered Regularly Interspaced Short Palindromic Repeats Activation; DZIP, Deleted in Azoospermia Interacting Zinc Finger Protein; Env, Envelope; ER, Endoplasmic Reticulum; ERGIC, Endoplasmic Reticulum-Golgi Intermediate Compartment; FCoV, Feline coronavirus; Gag-Pol, Group-specific antigen-polymerase; GBP, Guanylate-Binding Protein; GP, Gp or gp, Glycoprotein; GTP, Guanosine-5′-triphosphate; GWAS, Genome-wide association studies; HA, Hemagglutinin; HAND, HIV-Associated Neurocognitive Disorders; HAT, Human Airway Trypsin; HBEC, Human Bronchial Epithelial Cell; hCoV-229E, Human Coronavirus 229E; HCV, Hepatitis C Virus; HEK293, Human Embryonic Kidney 293 cells; HEK293T, Human Embryonic Kidney 293 cells expressing a mutant version of the SV40 large T antigen; HERV, Human Endogenous Retrovirus; HIV, Human Immunodeficiency Virus; hMPV, Human Metapneumovirus; HPAIV, Highly pathogenic avian influenza viruses; HPIV, Human Parainfluenzavirus; IAV, Influenza A virus; IFN, Interferon; IκB, Inhibitor of nuclear factor kappa B; KLK, Kallikrein-related peptidases; LasV, Lassa Virus; LTR, Long Terminal Repeat; MARCH, Membrane-Associated RING-CH-type finger; MERS-CoV, Middle East Respiratory Syndrome Coronavirus; MPI, Mucus Protease Inhibitor; Mpro, Main protease; NDV, Newcastle disease virus; NF-κB, Nuclear Factor kappa B; PACS, Phosphofurin Acidic Cluster Sorting Protein; PAI-1, Plasminogen Activator Inhibitor 1; PAR, Protease-Activated Receptor; PCSK, Proprotein Convertase Subtilisin/Kexin type; PLpro, Papain-like protease; pp1, Polyprotein 1; RCL, Reactive Center Loop; RSV, Respiratory Syncytial Virus; SARS-CoV-2, Severe Acute Respiratory Syndrome Coronavirus type 2; SERPIN, Serine Protease Inhibitor; SeV, Sendai Virus; SH, Small Hydrophobic; SLPI, Secretory Leukocyte Peptidase Inhibitor; SNAP, Soluble N-ethylmaleimide-Sensitive Factor Attachment Protein; SNARE, SNAP Receptors; SP, Surfactant Proteins; SPINK, Serine Peptidase or Protease Inhibitor Kazal type; TEV, Tobacco etch virus; TFLLR-NH2, Peptide derived from PAR-1; TMPRSS, Transmembrane Serine Protease; TNF, Tumor Necrosis Factor; Vpu, Viral Protein U; VSV, Vesicular Stomatitis Virus; WAP, Whey Acidic Protein.
